# PEARL: Pharmacy Education Applied to Resident Learners

**DOI:** 10.5811/westjem.2022.12.57219

**Published:** 2022-12-30

**Authors:** Jacob Lenning, Anna Nay, Matt Ogren, Bram Dolcourt, Kyle Mangan, Anne Messman

**Affiliations:** Wayne State University School of Medicine, Department of Emergency Medicine, Detroit, Michigan

## Abstract

**Introduction:**

Emergency medicine residents typically train with the support of emergency medicine pharmacists (EMP), but many EM residents will practice in post-graduation settings without EMP assistance. Therefore, a novel pharmacy curriculum for postgraduate year-1 (PGY-1) EMRs was developed, implemented, and assessed.

**Methods:**

We performed a controlled study of 25 residents from two separate EM programs in Detroit, MI. One program was the control group and the other program was the intervention group. The primary outcome was pre- and post-curriculum knowledge assessment scores, and the secondary outcome was pre- and post-curriculum, self-perceived knowledge survey responses. We performed statistical analyses with Welch’s t-test or the Mann-Whitney U test.

**Results:**

The pre-curriculum assessment scores (41% ± 11; 41% ± 8.1; P = 0.96; mean ± SD) and average pre-curriculum survey responses (2.8 ± 0.92; 3.0 ± 0.60; P = 0.35) were not statistically different between the control and the intervention groups. The post-curriculum assessment scores (63% ± 14; 74% ± 8.3; P = 0.04) and the average post-curriculum survey responses (4.2 ± 0.61; 5.0 ± 0.74, P = 0.02) were statistically different. The increase from the pre- to post-curriculum assessment scores (24% ± 11; 33% ± 11; P = 0.05) was also significantly different.

**Conclusion:**

The implementation of a novel pharmacy curriculum for PGY-1 EM residents resulted in improved knowledge of and comfort with pharmaceuticals and therapeutics specific to EM practice. The impact on patient care and frequency of medical errors requires further investigation.

## INTRODUCTION

Emergency medicine (EM) is a specialty centered on the diagnosis and management of a vast array of acute illnesses typically requiring the use of pharmacologic agents. This requires knowledge of proper indications, contraindications, mechanisms of action, drug-drug interactions, dosing, and methods of administration. The large scope of this information means EM pharmacists (EMP) are highly valued, and studies have shown that they contribute to improved patient care.^1^

Despite their proven value, EMP coverage in emergency departments (ED) is not universal. A 2015 national survey found that only two-thirds of EDs have EMP coverage for more than eight hours per day.^2^ Although EM residents have the advantage of training with EMPs in large academic settings, many residents will practice in post-graduation settings where the physician is responsible for many pharmacologic tasks.^2^ In an age of medicine wherein 19% of medical errors are drug-related, with 3% of these errors resulting in patient harm, it is imperative that EM residents are prepared to practice without the aid of EMPs.^3^,^4^

Unfortunately, the literature suggests that there are deficiencies in pharmacology knowledge among EM residents, especially during the early phases of training. One study surveyed first-year EM residents and found that only 8% rated their clinical pharmacology knowledge as “good,” while 30% rated it as “poor or worse” and the remainder as “average.”^5^ A separate study found that EM residents often have difficulty calculating dosages for common, life-saving medications.^6^ Such studies support the need for long-term interventions to reform EM education and prepare residents for post-training practice environments in which an EMP may not be immediately available. By doing so, medication errors and adverse drug events could be avoided.

The purpose of this intervention and investigation was to develop, implement, and evaluate a high-yield, longitudinal pharmacy curriculum for postgraduate year-1 (PGY-1) EM residents of the Wayne State University (WSU) EM residency at Sinai-Grace Hospital (SGH) of the Detroit Medical Center (DMC).

We used Kern’s six-step approach for medical education curriculum development.^7^ The identified problem was EM resident deficiency in the pharmacology knowledge required for EM practice. Assessment of the targeted needs of EM residents at SGH revealed that EMP presence in all resuscitations allowed the residents to mentally offload decisions of drug selection, dosage, and administration as the EMP tended to perform these tasks, especially for critical patients. Therefore, the overall goal of the curriculum was to teach EM residents basic pharmacological knowledge and tasks to prepare them for instances in which they may care for critical patients without pharmacist support post-graduation.

**Figure 1:**
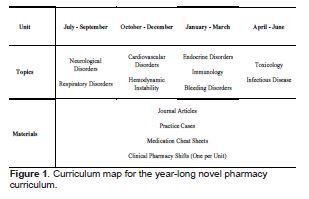
Curriculum map for the year-long novel pharmacy curriculum.

Specific objectives were formed by identifying common areas of deficiency in EM practice described in the literature and correlating these with the Accreditation Council for Graduate Medical Education (ACGME) core competencies and other specific learning objectives in the 2016 Model of Clinical Practice of EM.^8^ One multicenter study demonstrated that the most frequent medication errors by emergency physicians were associated with antimicrobial agents (32.1%), central nervous system medications (16.2%), anticoagulants and thrombolytics (12.7%), cardiovascular medications (6.7%), and hormonal agents (6.7%).^9^ The same study classified 29.1% of the reported medication errors as dosing errors, three times higher than any other type of error. A separate study of a confidential, online medication-error reporting system used by nearly 500 EDs across the country classified 18% of all medication errors as dosing errors, nearly twice the proportion of any other type of error.^3^ Considering these studies, we focused our objectives on mastering the dosing and administration of the most used and misused agents commonly used to treat the major diseases described in the ACGME core competencies. The objectives were divided into four units: 1) neurological and respiratory disorders; 2) cardiovascular disorders and hemodynamic instability, 3) endocrine, immunological, and bleeding disorders; and 4) toxicology and infectious disease (Figure 1).

**Population Health Research Capsule**

What do we already know about this issue?

*Emergency Medicine residents (EMRs) train with EM pharmacist (EMP) assistance, but often practice in post-graduations settings without EMP assistance.*

What was the research question?

*Does a year-long pharmacy curriculum for EMRs improve knowledge and comfort with pharmaceuticals and therapeutics?*

What was the major finding of the study?

*The post-curriculum knowledge assessments (p = 0.04) and knowledge surveys (p = 0.02) of the curriculum group were significantly improved compared to the non-curriculum group.*

How does this improve population health?

*This novel graduate medical education curriculum has potential to improve the quality and safety of medical care provided by emergency physicians.*

The educational strategies for meeting the objectives were influenced by Kolb’s experiential learning theory to foster knowledge acquisition through experience.^10^ Residents were provided with medication dosing charts, institutional guidelines, copies of landmark EM studies and review articles, and case-based learning modules for guided self-study during each three-month unit (Figure 1; Supplemental Materials). In addition, residents completed one-on-one clinical shifts with the EMP once per unit (Supplemental Materials). The medication dosing charts and institutional guidelines provided the factual knowledge for rapid review and quick reference. The landmark studies were selected to foster understanding of the reasons certain medications are favored in specific instances. Case-based learning modules allowed the residents to practice applying their knowledge. During clinical shifts, real-time patient encounters provided further practice for them to apply their knowledge. However, with the guidance of the EMP, higher levels of Bloom’s taxonomy were often reached as residents were led to analyze and evaluate new patient scenarios as they practiced making critical treatment decisions.^11^

The greatest barrier to implementation of the curriculum was finding time in the busy general EM curriculum for the residents to work directly with the EMP. The solution was to schedule the clinical pharmacy shifts for approximately four hours on a day that was already protected educational time and free from clinical responsibilities. Prior to full implementation of the curriculum, the clinical pharmacy shifts were trialed by senior residents. One adjustment made after the trial shifts included incorporating discussions led by the EMP pertaining to the specific unit, especially if the patient encounters that day were sparse. Moreover, the EMP was prepared to guide the residents through hands-on-tasks such as preparing and administering specific medications.

Once the curriculum was developed and implemented, we considered Kirkpatrick’s model to evaluate the ability of the curriculum to meet the defined purpose and objectives. Per the model, a training or educational program can be evaluated at four possible levels: 1) the learner’s reaction; 2) improvement in knowledge; 3) change in behavior; and 4) impact on patient outcomes.^12^ For the purposes of this investigation, the aim was to achieve first- and second-level outcomes as measured by assessment of the participants’ comfort level with the material of the curriculum and of the participants’ level of knowledge after completing the curriculum.

## METHODS

### Study Design

This prospective, repeated-measure, controlled study was reviewed by the WSU Institutional Review Board and deemed exempt. The study was conducted from June 2020–July 2021 in the WSU Department of Emergency Medicine encompassing two separate DMC EM residency programs: Sinai-Grace Hospital (SGH), an urban/community center, was used as the intervention site; and Detroit Receiving Hospital (DRH), an urban/academic center, was used as the control site.

Notably, both residency programs are staffed by the same physician group. Both hospitals have the same policies, procedures, guidelines, and formulary management from both a physician and pharmacist standpoint. Each ED has a satellite pharmacy that is staffed 24/7 by a pharmacist. Furthermore, each ED has an American Society of Health-System Pharmacists (ASHP) PGY-2 residency-trained EM pharmacy specialist who is solely dedicated to working in the ED. The pharmacists’ roles, involvement, and expectations are the same at both hospitals. Pharmacists at both institutions attend all medical and trauma resuscitations, intubations, and procedural sedations. Additionally, the pharmacists have the same responsibilities regarding order verification, dosing consults, and intravenous medication compounding.

### Participants

A total of 25 WSU PGY-1 EM residents from SGH (12) and DRH (13) participated in the study. Written consent was obtained. Demographics of the participants were not recorded. The control group consisted of the 13 PGY-1 EM residents at DRH who followed the standard EM residency curriculum without the additional pharmacy curriculum. The intervention group consisted of the 12 PGY-1 EM residents at SGH who completed the novel pharmacy curriculum in addition to the standard EM residency curriculum.

### Outcome Measures and Data Collection

The primary outcome was the difference in scores of an identical pre- and post-curriculum knowledge assessment, which consisted of a case-based, 30-question multiple-choice examination. Scores were tabulated as the percentage of questions correct. The questions were written by the resident authors and the EMP. The examination was proctored over one hour by WSU EM faculty. The pre-curriculum assessment was conducted in July 2020 and the post-curriculum assessment in July 2021. Each assessment was completed individually and anonymously by the participants without access to supplemental resources.

The secondary outcome was the difference in average values of an identical pre- and post-curriculum self-perceived knowledge survey, which consisted of eight questions to elicit self-perceived knowledge and comfort with pharmacology and therapeutics. Responses were based on a seven-point Likert scale. We tabulated average values for the survey. The questions of the assessments and the surveys were content-validated by WSU EM faculty and then piloted by residents who were not participating in the study.

### Data Analysis

We used RStudio (Boston, MA) software for statistical analysis. Mean and standard deviation for each dataset were calculated. We assessed normality with histogram plots and with the Shapiro-Wilk test. The datasets of the pre- and post-curriculum knowledge assessments for both the control and intervention groups followed normal distributions based on visual analysis of the histograms and the universally non-significant *P*-values of the Shapiro-Wilk test. Therefore, we performed Welch’s two sample, two-tailed *t*-tests to compare assessment scores. The datasets of the pre- and post-curriculum self-perceived knowledge surveys for both the control and intervention groups did not universally follow normal distributions as evidenced by skewed histograms and significant *P*-values reported by the Shapiro-Wilk test. Therefore, Mann-Whitney U tests were performed to compare the average survey responses. The level of significance α = 0.05 was assumed for all statistical testing.

## RESULTS

In total, 25 PGY-1 EM residents participated in the study, 13 in the control group and 12 in the intervention group. Two participants in the control group did not complete the post-curriculum knowledge assessment and five participants in the control group did not complete the post-curriculum, self-perceived knowledge survey. One participant in the intervention group did not complete the pre-curriculum, self-perceived knowledge survey. The participants with missing responses were only excluded from the comparison of the differences between the pre- and post-curriculum knowledge assessments and self-perceived knowledge surveys within the treatment groups. However, these participants were otherwise included in the comparison of the knowledge assessment percentage scores and the average self-perceived knowledge survey responses between the two groups.

### Assessment Scores

**Figure 2:**
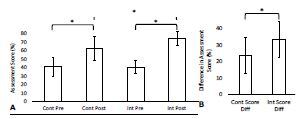
(A) Mean ± SD (error bars) of the percent correct of the pre- and post-curriculum assessments for the control group and intervention group. (B) Mean ± SD of the differences between the percent correct of the pre- and post-curriculum assessments for the control group and intervention group. Asterisks (*) denote P-values representative of statistically significant differences between the control group and the intervention group. Level of significance α = 0.05 was assumed. P-values are from Welch’s two-sample t-tests performed to compare the normally distributed assessment data. *Cont*, control; *Int*, intervention; *Diff*, difference

The baseline pre-curriculum knowledge assessment scores were not significantly different between the two groups with the control group averaging 41% ± 11; n = 13 (mean ± standard deviation; number of participants) and the intervention group averaging 41% ± 8.1; n = 12 (*P* = 0.96). The post-curriculum knowledge assessment scores were significantly different with the control group averaging 63% ± 14; n = 11 and the intervention group averaging 74% ± 8.3; n = 12 (*P* = 0.04). The pre- and post-curriculum knowledge assessment scores were significantly different for both the control group (*P* < 0.01) and the intervention group (*P* < 0.01). The differences between the pre- and post-curriculum knowledge assessment scores were significantly different with the control group averaging an increase of 24% ± 11; n = 11, and the intervention group averaging an increase of 33% ± 11; n = 12 (*P* = 0.05) (Table 1, Figure 2).

### Average Survey Values

**Figure 3:**
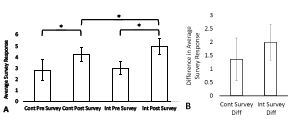
(A) Mean ± SD of the average values of the pre- and post-curriculum self-perceived knowledge surveys for the control group and intervention group. (B) Mean ± SD of the differences between the average values of the pre- and post-curriculum, self-perceived knowledge surveys for the control group and intervention group. Asterisks (*) denote P-values representative of statistically significant differences between the control group and the intervention group. Level of significance α = 0.05 was assumed. P-values are from the Mann-Whitney U tests performed to compare the skewed survey data. *Cont*, control; *Int*, intervention; *Diff*, difference

The baseline average pre-curriculum, self-perceived knowledge survey responses were not significantly different with the control group averaging 2.8 ± 0.92; n = 13 (mean ± SD; number of participants) and the intervention group averaging 3.0 ± 0.60; n = 11 (*P* = 0.35). The average post-curriculum, self-perceived knowledge survey responses were significantly different with the control group averaging 4.2 ± 0.61; n = 8 and the intervention group averaging 5.0 ± 0.74; n = 12 (*P* = 0.02). The average pre- and post-curriculum self-perceived knowledge survey responses were significantly different for both the control group (*P* < 0.01) and the intervention group (*P* < 0.01). The differences between the average pre- and post-curriculum, self-perceived knowledge survey responses were not significantly different with the control group averaging an increase of 1.4 ± 0.79; n = 8, and the intervention group averaging an increase of 1.9 ± 0.85; n = 11 (*P* = 0.20). (Table 1, Figure 3)

## DISCUSSION

This controlled study demonstrated the benefit of a novel pharmacy curriculum for PGY-1 EM residents as indicated by a significantly larger improvement in pre- to post-curriculum knowledge-assessment scores (33% and 24%; *P* = 0.05) in the intervention group compared to the control group. Importantly, the mean of the pre-curriculum knowledge-assessment scores was the same for both groups (41%; *P* = 0.96) and the mean of the average pre-curriculum, self-perceived knowledge survey responses was also very similar for the intervention and the control groups (3.0 and 2.8, *P* = 0.35). These results established that both groups began the study period with a similar knowledge base and comfort level regarding EM pharmacology. This allowed for direct comparison of the post-curriculum knowledge-assessment scores and the post-curriculum, self-perceived knowledge survey responses between the control and intervention groups.

As expected, knowledge assessment scores and self-perceived knowledge survey responses increased over time in both groups, but the intervention group demonstrated greater improvement. Although the increase from the average pre- to post-curriculum, self-perceived knowledge survey responses was not significantly different between the intervention and the control group (1.9 and 1.4; *P* = 0.20), the average post-curriculum, self-perceived knowledge survey responses were significantly different between groups (5.0 and 4.2; *P* = 0.02). Therefore, the results still suggest that the intervention group finished the curriculum with a higher level of self-perceived knowledge of the material than the control group. Overall, the results suggest that the intervention group gained additional knowledge and that their comfort level increased because of the curriculum.

The strengths of this study are its longitudinal nature and controlled design. The curriculum was delivered over an entire academic year, which provided enough time to cover the most frequently used medications for the most common EM patient presentations in a systematic and organ system-based approach. While previous studies have already demonstrated the benefit of short-term interventions,^5^,^14^ we could not identify any studies in our literature review that had implemented and investigated such an extensive and comprehensive curriculum.

One short-term study focused on the appropriate calculation of doses for select medications and concluded that a single, brief education session led to short-term improvement in EM resident performance of such tasks.^5^ However, the reassessment was only six weeks after the educational session, and long-term knowledge retention was not assessed. Furthermore, the control group did not perform the pre-assessment; therefore, it was unknown whether there were baseline differences between the control group and the intervention group. Considering this, our study was specifically designed to account for possible baseline differences between the control and intervention groups by administering the pre-curriculum knowledge assessment to each group. Moreover, the pre- and post-curriculum knowledge assessments were separated by an entire calendar year to assess long-term knowledge retention of the material presented in the curriculum.

The positive results of our study in combination with insights from previous literature suggest that novel curricula like ours may have the potential to impact patient-centered outcomes. In one previous study, pediatric emergency resident rotators participated in didactic sessions and daily discussions regarding the best practices of medication administration with an EMP and attending physicians over the course of one month.^14^ Medication dosing errors and adverse events were significantly less frequent after implementation of the program. Considering that our novel curriculum was more comprehensive than the short-term curriculum used in the study of pediatric emergency resident rotators, one could speculate that our novel curriculum could lead to improvement in patient outcomes. Further studies investigating clinical and patient-centered outcomes, such as number of incorrect medication orders and medication administration errors before and after implementation of a similar pharmacy curriculum, should be conducted.

There is already extensive literature demonstrating that EMPs improve patient care and reduce medical errors. However, the role of EMPs in EM resident physician education is less defined. The ASHP guidelines on EMP service recommend that EMPs play an active role in interdisciplinary education among other healthcare professionals. The opportunity for such education is broad, and the guidelines do not specify in what capacity an EMP should educate; rather, the guidelines nonspecifically encourage involvement in formal and informal education.^15^ Our novel curriculum classifies as interdisciplinary education and demonstrates the value of an EMP while specifically outlining methods in which an EMP may positively impact resident physician knowledge. In doing so, our study helps define the role of EMPs in optimizing and improving EM resident physician knowledge in ways that could translate into improved patient outcomes.

## LIMITATIONS

The first limitation has to do with the study design in which the control group and intervention group were PGY-1 classes from two separate EM residency programs. Ideally, the residents would have been blindly randomized into the control and intervention groups, however, splitting the residents from the same program among the intervention and control groups would have been difficult and of questionable ethics. Therefore, despite the same physician group staffing both EDs and despite very similar pharmacy services being provided at both institutions, the possibility exists that the SGH residency program assigned to the intervention group may have had a more robust pharmacy education at baseline to account for the results of the study.

The second limitation was the rather small sample size, which was made smaller by the number of post-curriculum knowledge assessments and post-curriculum self-perceived knowledge surveys that were not completed, leading to large variance in the datasets. Likely, a larger study population with improved follow-up would have lessened the variance. The study was also limited by the available number of PGY-1 EM residents and could not be designed with an appropriate sample size for the estimated effect size. Despite achieving statistical significance, the effective increase in assessment scores and average survey responses was still small and, therefore, the possibility of a type I error still exists.

The purpose of the study was simply to support adoption of the novel pharmacy curriculum; and so the statistical results are less important than the educational and clinical significance. Logically, the introduction of a pharmacy curriculum should increase participant knowledge and self-perceived knowledge. Therefore, any positive results from the limited sample size support implementation of the curriculum because the potential benefits in resident education and clinical patient care far outweigh any potential risks of implementation.

Perhaps the largest limitation of our study is that the primary outcome—change in knowledge-assessment scores following the intervention—is a second level outcome in Kirkpatrick’s model.^9^ The inspiration for development of the novel pharmacy curriculum was to prepare EM residents for post-graduation practice without EMP assistance and reduce medication errors to improve patient care. While these fourth-level outcomes of Kirkpatrick’s model were not feasible to measure in this study, it would be interesting and important to investigate in future studies.

## CONCLUSION

The implementation of a novel pharmacy curriculum for first-year EM residents resulted in improved knowledge of and comfort with pharmaceuticals and therapeutics specific to EM practice. The impact on patient care and frequency of medical errors requires further investigation.

## Supplementary Information









## Figures and Tables

**Table:** Comparison of assessment scores and survey responses.

	Control group (χ̄ ± SD)	Intervention group (χ̄ ± SD)	*P*-value
Pre-assessment score	41 ± 11 n = 13	41 ± 8.1 n = 12	0.96
Post-assessment score	63 ± 14 n = 11	74 ± 8.3 n = 12	0.04^*^
Difference in assessment score	24 ± 11 n = 11	33 ± 11 n = 12	0.05^*^
Average pre-survey response	2.8 ± 0.92 n = 13	3.0 ± 0.60 n = 11	0.35
Average post-survey response	4.2 ± 0.61 n = 8	5.0 ± 0.74 n = 12	0.02^*^
Difference in survey response	1.4 ± 0.79 n = 8	1.9 ± 0.85 n = 11	0.20

*χ̄*, mean; *SD*, standard deviation; *n*, number of participants.
