# Supplementary material for: PEARL: Pharmacy Education Applied to Resident Learners
Source: West J Emerg Med. 2022 Dec 30;24(1):23–9. doi: 10.5811/westjem.2022.12.57219 (PMC9897259; doi:10.5811/westjem.2022.12.57219)
Supplement: Supplementary file 1 [file wjem-24-23-s001.docx]

**CDEM/CORD Education Special Issue**

**PEARL: PHARMACY EDUCATION APPLIED TO RESIDENT LEARNERS**

**Pharmacy Shift Tasks and Objectives**

Examples of tasks and objectives performed by emergency medicine PGY-1 residents during the once per unit (every 3 months) clinical shifts (each last 4 hours) with the emergency medicine pharmacy specialist.

**Objectives:**

- Discuss landmark journal articles.
- Discuss questions about practice cases from self-guided study.
- Review medications available in the crash carts.
- Review available institutional guidelines.
- Discuss pharmacy resources ie, Lexicomp.

**Tasks:**

- Mixing push-dose pressors.
- Observe and understand IV admixture.
- Understand operational pharmacy
  - Order verification, medication preparation, medication delivery.
- Attend all medical and trauma codes with debrief regarding medications, dosing, and routes utilized.
