# Supplementary material for: PEARL: Pharmacy Education Applied to Resident Learners
Source: West J Emerg Med. 2022 Dec 30;24(1):23–9. doi: 10.5811/westjem.2022.12.57219 (PMC9897259; doi:10.5811/westjem.2022.12.57219)
Supplement: Supplementary file 2 [file wjem-24-23-s002.docx]

**CDEM/CORD Education Special Issue**

**PEARL: PHARMACY EDUCATION APPLIED TO RESIDENT LEARNERS**

**Curriculum References**

**Unit 1**

Neurological Disorders

- Excited Delirium
  - Riddell J, Tran A, Bengiamin R, Hendey GW, Armenian P. Ketamine as a first-line treatment for severely agitated emergency department patients. Am J Emerg Med. 2017 Jul;35(7):1000-1004.
  - Wilson MP, Pepper D, Currier GW, Holloman GH Jr, Feifel D. The psychopharmacology of agitation: consensus statement of the american association for emergency psychiatry project Beta psychopharmacology workgroup. West J Emerg Med. 2012 Feb;13(1):26-34.
- Ischemic Stroke
  - National Institute of Neurological Disorders and Stroke rt-PA Stroke Study Group. Tissue plasminogen activator for acute ischemic stroke. N Engl J Med. 1995 Dec 14;333(24):1581-7.
  - Hacke W, Kaste M, Bluhmki E, Brozman M, Dávalos A, Guidetti D, Larrue V, Lees KR, Medeghri Z, Machnig T, Schneider D, von Kummer R, Wahlgren N, Toni D; ECASS Investigators. Thrombolysis with alteplase 3 to 4.5 hours after acute ischemic stroke. N Engl J Med. 2008 Sep 25;359(13):1317-29.
- Procedural Sedation
  - Tobias JD, Leder M. Procedural sedation: A review of sedative agents, monitoring, and management of complications. Saudi J Anaesth. 2011 Oct;5(4):395-410.
- Status Epilepticus
  - Glauser T, Shinnar S, Gloss D, Alldredge B, Arya R, Bainbridge J, Bare M, Bleck T, Dodson WE, Garrity L, Jagoda A, Lowenstein D, Pellock J, Riviello J, Sloan E, Treiman DM. Evidence-Based Guideline: Treatment of Convulsive Status Epilepticus in Children and Adults: Report of the Guideline Committee of the American Epilepsy Society. Epilepsy Curr. 2016 Jan-Feb;16(1):48-61.
  - Braun J, Gau E, Revelle S, Byrne L, Kumar A. Impact of non-guideline-based treatment of status epilepticus. J Neurol Sci. 2017 Nov 15;382:126-130.
  - Kapur J, Elm J, Chamberlain JM, Barsan W, Cloyd J, Lowenstein D, Shinnar S, Conwit R, Meinzer C, Cock H, Fountain N, Connor JT, Silbergleit R; NETT and PECARN Investigators. Randomized Trial of Three Anticonvulsant Medications for Status Epilepticus. N Engl J Med. 2019 Nov 28;381(22):2103-2113.
  - Kapur J, Elm J, Chamberlain JM, Barsan W, Cloyd J, Lowenstein D, Shinnar S, Conwit R, Meinzer C, Cock H, Fountain N, Connor JT, Silbergleit R; NETT and PECARN Investigators. Randomized Trial of Three Anticonvulsant Medications for Status Epilepticus. N Engl J Med. 2019 Nov 28;381(22):2103-2113.
  - Silbergleit R, Durkalski V, Lowenstein D, Conwit R, Pancioli A, Palesch Y, Barsan W; NETT Investigators. Intramuscular versus intravenous therapy for prehospital status epilepticus. N Engl J Med. 2012 Feb 16;366(7):591-600.
  - Brophy GM, Bell R, Claassen J, Alldredge B, Bleck TP, Glauser T, Laroche SM, Riviello JJ Jr, Shutter L, Sperling MR, Treiman DM, Vespa PM; Neurocritical Care Society Status Epilepticus Guideline Writing Committee. Guidelines for the evaluation and management of status epilepticus. Neurocrit Care. 2012 Aug;17(1):3-23.

Respiratory Disorders

- Angioedema
  - Bernstein JA, Cremonesi P, Hoffmann TK, Hollingsworth J. Angioedema in the emergency department: a practical guide to differential diagnosis and management. Int J Emerg Med. 2017 Dec;10(1):15.
- Asthma
  - Camargo CA Jr, Rachelefsky G, Schatz M. Managing asthma exacerbations in the emergency department: summary of the National Asthma Education and Prevention Program Expert Panel Report 3 guidelines for the management of asthma exacerbations. J Emerg Med. 2009 Aug;37(2 Suppl):S6-S17.
  - Lazarus SC. Clinical practice. Emergency treatment of asthma. N Engl J Med. 2010 Aug 19;363(8):755-64.
- COPD
  - Leuppi JD, Schuetz P, Bingisser R, Bodmer M, Briel M, Drescher T, Duerring U, Henzen C, Leibbrandt Y, Maier S, Miedinger D, Müller B, Scherr A, Schindler C, Stoeckli R, Viatte S, von Garnier C, Tamm M, Rutishauser J. Short-term vs conventional glucocorticoid therapy in acute exacerbations of chronic obstructive pulmonary disease: the REDUCE randomized clinical trial. JAMA. 2013 Jun 5;309(21):2223-31.
  - Singh D, Agusti A, Anzueto A, Barnes PJ, Bourbeau J, Celli BR, Criner GJ, Frith P, Halpin DMG, Han M, López Varela MV, Martinez F, Montes de Oca M, Papi A, Pavord ID, Roche N, Sin DD, Stockley R, Vestbo J, Wedzicha JA, Vogelmeier C. Global Strategy for the Diagnosis, Management, and Prevention of Chronic Obstructive Lung Disease: the GOLD science committee report 2019. Eur Respir J. 2019 May 18;53(5):1900164.
  - Kiser TH, Allen RR, Valuck RJ, Moss M, Vandivier RW. Outcomes associated with corticosteroid dosage in critically ill patients with acute exacerbations of chronic obstructive pulmonary disease. Am J Respir Crit Care Med. 2014 May 1;189(9):1052-64.
- RSI
  - Stephens RJ, Dettmer MR, Roberts BW, Ablordeppey E, Fowler SA, Kollef MH, Fuller BM. Practice Patterns and Outcomes Associated With Early Sedation Depth in Mechanically Ventilated Patients: A Systematic Review and Meta-Analysis. Crit Care Med. 2018 Mar;46(3):471-479.
  - Mason MA, Weant KA, Baker SN. Rapid sequence intubation medication therapies: a review in light of recent drug shortages. Adv Emerg Nurs J. 2013 Jan-Mar;35(1):16-25.

**Unit 2**

Cardiovascular Disorders

- Pulmonary Embolism
  - Marti C, John G, Konstantinides S, Combescure C, Sanchez O, Lankeit M, Meyer G, Perrier A. Systemic thrombolytic therapy for acute pulmonary embolism: a systematic review and meta-analysis. Eur Heart J. 2015 Mar 7;36(10):605-14.
  - Gouin B, Robert-Ebadi H, Righini M, Blondon M. Pharmacological management of pulmonary embolism. Expert Opin Pharmacother. 2017 Jan;18(1):79-93.
  - Sharifi M, Berger J, Beeston P, Bay C, Vajo Z, Javadpoor S; “PEAPETT” investigators. Pulseless electrical activity in pulmonary embolism treated with thrombolysis (from the "PEAPETT" study). Am J Emerg Med. 2016 Oct;34(10):1963-1967.
  - Sharifi M, Bay C, Skrocki L, Rahimi F, Mehdipour M; “MOPETT” Investigators. Moderate pulmonary embolism treated with thrombolysis (from the "MOPETT" Trial). Am J Cardiol. 2013 Jan 15;111(2):273-7.
- Heart Failure
  - Wilson SS, Kwiatkowski GM, Millis SR, Purakal JD, Mahajan AP, Levy PD. Use of nitroglycerin by bolus prevents intensive care unit admission in patients with acute hypertensive heart failure. Am J Emerg Med. 2017 Jan;35(1):126-131.
  - Levy P, Compton S, Welch R, Delgado G, Jennett A, Penugonda N, Dunne R, Zalenski R. Treatment of severe decompensated heart failure with high-dose intravenous nitroglycerin: a feasibility and outcome analysis. Ann Emerg Med. 2007 Aug;50(2):144-52.
- AAA / Aortic Dissection
  - Yoshimura K, Morikage N, Nishino-Fujimoto S, Furutani A, Shirasawa B, Hamano K. Current Status and Perspectives on Pharmacologic Therapy for Abdominal Aortic Aneurysm. Curr Drug Targets. 2018;19(11):1265-1275.
  - Yang HJ, Kim JG, Lim YS, Ryoo E, Hyun SY, Lee G. Nicardipine versus nitroprusside infusion as antihypertensive therapy in hypertensive emergencies. J Int Med Res. 2004 Mar-Apr;32(2):118-23.
  - Suzuki T, Eagle KA, Bossone E, Ballotta A, Froehlich JB, Isselbacher EM. Medical management in type B aortic dissection. Ann Cardiothorac Surg. 2014 Jul;3(4):413-7.

Hemodynamic Instability

- Shock
  - Hollenberg SM. Inotrope and vasopressor therapy of septic shock. Crit Care Clin. 2009 Oct;25(4):781-802, ix.
  - Overgaard CB, Dzavík V. Inotropes and vasopressors: review of physiology and clinical use in cardiovascular disease. Circulation. 2008 Sep 2;118(10):1047-56.
- Cardiopulmonary Arrest
  - Perkins GD, Ji C, Deakin CD, Quinn T, Nolan JP, Scomparin C, Regan S, Long J, Slowther A, Pocock H, Black JJM, Moore F, Fothergill RT, Rees N, O'Shea L, Docherty M, Gunson I, Han K, Charlton K, Finn J, Petrou S, Stallard N, Gates S, Lall R; PARAMEDIC2 Collaborators. A Randomized Trial of Epinephrine in Out-of-Hospital Cardiac Arrest. N Engl J Med. 2018 Aug 23;379(8):711-721.
  - Papastylianou A, Mentzelopoulos S. Current pharmacological advances in the treatment of cardiac arrest. Emerg Med Int. 2012;2012:815857.

**Unit 3**

Endocrine Disorders

- Diabetes
  - Karslioglu French E, Donihi AC, Korytkowski MT. Diabetic ketoacidosis and hyperosmolar hyperglycemic syndrome: review of acute decompensated diabetes in adult patients. BMJ. 2019 May 29;365:l1114. doi: 10.1136/bmj.l1114. PMID: 31142480.
- Thyroid
  - Idrose AM. Acute and emergency care for thyrotoxicosis and thyroid storm. Acute Med Surg. 2015 May 12;2(3):147-157.
  - Kwaku MP, Burman KD. Myxedema coma. J Intensive Care Med. 2007 Jul-Aug;22(4):224-31.
- Glaucoma
  - Murray D. Emergency management: angle-closure glaucoma. Community Eye Health. 2018;31(103):64. PMID: 30487684; PMCID: PMC6253313.

Immunology

- Anaphylaxis (institutional guidelines)

Bleeding Disorders

- TBI
  - CRASH-3 trial collaborators. Effects of tranexamic acid on death, disability, vascular occlusive events and other morbidities in patients with acute traumatic brain injury (CRASH-3): a randomised, placebo-controlled trial. Lancet. 2019 Nov 9;394(10210):1713-1723. doi: 10.1016/S0140-6736(19)32233-0. Epub 2019 Oct 14. Erratum in: Lancet. 2019 Nov 9;394(10210):1712.
  - CRASH-2 trial collaborators, Shakur H, Roberts I, Bautista R, Caballero J, Coats T, Dewan Y, El-Sayed H, Gogichaishvili T, Gupta S, Herrera J, Hunt B, Iribhogbe P, Izurieta M, Khamis H, Komolafe E, Marrero MA, Mejía-Mantilla J, Miranda J, Morales C, Olaomi O, Olldashi F, Perel P, Peto R, Ramana PV, Ravi RR, Yutthakasemsunt S. Effects of tranexamic acid on death, vascular occlusive events, and blood transfusion in trauma patients with significant haemorrhage (CRASH-2): a randomised, placebo-controlled trial. Lancet. 2010 Jul 3;376(9734):23-32.
- Intracranial Hemorrhage
  - Anderson CS, Heeley E, Huang Y, Wang J, Stapf C, Delcourt C, Lindley R, Robinson T, Lavados P, Neal B, Hata J, Arima H, Parsons M, Li Y, Wang J, Heritier S, Li Q, Woodward M, Simes RJ, Davis SM, Chalmers J; INTERACT2 Investigators. Rapid blood-pressure lowering in patients with acute intracerebral hemorrhage. N Engl J Med. 2013 Jun 20;368(25):2355-65.
  - Arima H, Heeley E, Delcourt C, Hirakawa Y, Wang X, Woodward M, Robinson T, Stapf C, Parsons M, Lavados PM, Huang Y, Wang J, Chalmers J, Anderson CS; INTERACT2 Investigators; INTERACT2 Investigators. Optimal achieved blood pressure in acute intracerebral hemorrhage: INTERACT2. Neurology. 2015 Feb 3;84(5):464-71.
  - Hemphill JC 3rd, Greenberg SM, Anderson CS, Becker K, Bendok BR, Cushman M, Fung GL, Goldstein JN, Macdonald RL, Mitchell PH, Scott PA, Selim MH, Woo D; American Heart Association Stroke Council; Council on Cardiovascular and Stroke Nursing; Council on Clinical Cardiology. Guidelines for the Management of Spontaneous Intracerebral Hemorrhage: A Guideline for Healthcare Professionals From the American Heart Association/American Stroke Association. Stroke. 2015 Jul;46(7):2032-60.
- Epistaxis
  - Joseph J, Martinez-Devesa P, Bellorini J, Burton MJ. Tranexamic acid for patients with nasal haemorrhage (epistaxis). Cochrane Database Syst Rev. 2018 Dec 31;12(12):CD004328.
- Disseminated Intravascular Coagulation
  - Levi M, Toh CH, Thachil J, Watson HG. Guidelines for the diagnosis and management of disseminated intravascular coagulation. British Committee for Standards in Haematology. Br J Haematol. 2009 Apr;145(1):24-33.
- Anticoagulation Reversal
  - Lubetsky A, Yonath H, Olchovsky D, Loebstein R, Halkin H, Ezra D. Comparison of oral vs intravenous phytonadione (vitamin K1) in patients with excessive anticoagulation: a prospective randomized controlled study. Arch Intern Med. 2003 Nov 10;163(20):2469-73.

**Unit 4**

Toxicology

- Gallagher N, Edwards FJ. The Diagnosis and Management of Toxic Alcohol Poisoning in the Emergency Department: A Review Article. Adv J Emerg Med. 2019 May 22;3(3):e28.
- Wermeling DP. Review of naloxone safety for opioid overdose: practical considerations for new technology and expanded public access. Ther Adv Drug Saf. 2015 Feb;6(1):20-31. doi: 10.1177/2042098614564776. PMID: 25642320; PMCID: PMC4308412.
- Arens AM, Shah K, Al-Abri S, Olson KR, Kearney T. Safety and effectiveness of physostigmine: a 10-year retrospective review. Clin Toxicol (Phila). 2018 Feb;56(2):101-107.
- Beta / Calcium Blocker Overdose (institutional guidelines)
- Acetaminophen Overdose (institutional guidelines)
- Salicylate Overdose (institutional guidelines)

Infectious Disease

- Sepsis (institutional guidelines)
  - Howell MD, Davis AM. Management of Sepsis and Septic Shock. JAMA. 2017;317(8):847–848. doi:10.1001/jama.2017.0131
- Rabies
  - World Health Organization. Rabies vaccines: WHO position paper, April 2018 - Recommendations. Vaccine. 2018 Sep 5;36(37):5500-5503. doi: 10.1016/j.vaccine.2018.06.061. Epub 2018 Aug 11. PMID: 30107991.
- HIV (institutional guidelines)
- Tetanus (institutional guidelines)
- Antibiotic Stewardship (institutional guidelines)
