# Supplementary material for: PEARL: Pharmacy Education Applied to Resident Learners
Source: West J Emerg Med. 2022 Dec 30;24(1):23–9. doi: 10.5811/westjem.2022.12.57219 (PMC9897259; doi:10.5811/westjem.2022.12.57219)
Supplement: Supplementary file 3 [file wjem-24-23-s003.docx]

**Module 1 Medication Quick Reference**

**Nervous System Disorders**

**Status Epilepticus**

- Midazolam (Versed) 5-10 mg IM/IV (peds 0.2 mg/kg)
- Lorazepam (Ativan) 2-4 mg IV (peds 0.1 mg/kg)
  - Levetiracetam (Keppra) Loading dose 60 mg/kg, maximum 4500 mg
    - Propofol (Diprivan) 1-2 mg/kg IV loading, then infusion of 5-50 mcg/kg/min
      - Phenobarbital 15-20 mg/kg IV loading at 30-50 mg/min

**Ischemic Stroke**

- tPA 0.9 mg/kg (max 90 mg). 10% given as IV bolus ~1 min. 90% infused over 1 hour.

**Excited Delirium**

- Ketamine 4 mg/kg IM, OR
- Midazolam (Versed) 2-5 mg IM, OR
- Haloperidol (Haldol) 2.5-5 mg IM
- If secondary to psychiatric illness consider using
  - Haloperidol (Haldol) 2.5-5 mg IM
  - Olanzapine (Zyprexa) 10 mg IV
- Consider adding benzodiazepines as well (Midazolam 2-5 mg IV; Lorazepam 2-4 mg IV) if required

**Procedural Sedation**

- Ketamine 0.5-1 mg/kg
- Propofol 0.5-1 mg/kg
- Fentanyl 1-2 mcg/kg + Versed 0.05 - 0.1 mg/kg
- Etomidate 0.1-0.2 mg/kg

**Respiratory Disorders**

**RSI Medications**

- Induction agents
  - Etomidate 0.3 mg/kg IV (standard adult: 20mg)
  - Ketamine 1-2 mg/kg IV (standard adult: 100mg)
  - Propofol (Diprivan) 1-2 mg/kg IV (standard adult: 140mg)
  - Midazolam (Versed) 0.3 mg/kg IV (standard adult: 20mg)
- Paralytics
  - Succinylcholine 1.0-1.5 mg/kg IV (standard adult: 100mg)
  - Rocuronium 0.6-1.2 mg/kg IV (standard adult: 70 mg)
  - Vecuronium 0.1-0.2 mg/kg IV (standard adult: 10 mg)
  - Cisatracurium 0.1-0.2 mg/kg IV (standard adult: 10 mg)

**Post-Intubation Sedation**

- Fentanyl: Bolus 1-3 mcg/kg, Drip 1-10 mcg/kg/hr
- Propofol: Bolus 0.5-1 mg/kg, Drip 5-50 mcg/kg/min
- Midazolam: Bolus 1-5 mg, Drip 2-15 mg/hr

**Angioedema**

- Epinephrine 0.3mg IM (1:1000)
- Diphenhydramine (Benadryl) 50 mg IV
- Methylprednisolone (Solu-Medrol) 125 mg IV
- Famotidine (Pepcid) 20 mg IV (0.25 mg/kg)
  - Icatibant 30 mg SQ if hereditary angioedema

**Asthma**

- Albuterol 5mg/Ipratropium bromide 0.5 mg (“5/.5”) NEB q20min x3 doses
- Methylprednisolone (Solu-Medrol) 40 mg IV
  - Peds use dexamethasone 0.6 mg/kg max 10 mg
- Magnesium 25-70 mg/kg IV over 30 min (standard adult: 2g)
- Epinephrine 0.3-0.5 mg IM (1:1000)
  - Peds 0.01 mg/kg
- Terbutaline 0.25 mg SQ
  - Peds 0.01 mg/kg

**COPD**

- Albuterol 5 mg/Ipratropium bromide 0.5 mg (“5/.5”) NEB q20min x3 doses
- Methylprednisolone (Solu-Medrol) 40 mg IV
- Magnesium 25-70 mg/kg IV over 30 min (standard adult: 2 g)

**Module 2 Medication Quick Reference**

**Decompensated Heart Failure**

- Nitroglycerin
  - 1-2 mg IVP bolus q3-5 minutes (max 20 mg)
  - If continuous infusion needed, range 5-200mcg/min
    - In this setting, start at 100mcg/min
- Furosemide (Lasix) 0.5-1.0 mg/kg IV

**Pulmonary Embolism**

- Thrombolysis
  - tPA 100 mg IV over 2 hours
- Anticoagulation
  - Heparin 80 units/kg bolus then 18 units/kg/hr
  - Enoxaparin (Lovenox) 1 mg/kg SC q12h

**SVT**

- Adenosine 6 mg rapid IV bolus; If needed repeat 12 mg rapid IV bolus (X2)
- Diltiazem (Cardizem) 0.25 - 0.35 mg/kg IV bolus over 2 minutes
  - Typical starting dose 20mg; repeat 25mg

**Sustained V Tach**

- Procainamide 17 mg/kg (max 1gm; infuse 50mg/min) then infusion of 1-4 mg/min (max 6mg/min) for 6 hours.
- Amiodarone 150 mg over 10 min followed by 1 mg/min drip over 6 hours

**Hyperkalemia**

- Calcium Gluconate 1-2 gm (over 5-10 minutes); repeat if needed
- Insulin and Glucose 10 units regular insulin and 50-100 mL (1-2 amps) of D50
- Albuterol 10-20 mg nebulized over 20 minutes
- Bicarbonate 50 mEq (1 amp)
- Lasix 40 mg IV

**Cardiogenic Shock**

- Norepinephrine (Levophed) 0.1 mcg/kg/min infusion
- Dobutamine 2-20 mcg/kg/min infusion
- Milrinone loading dose 50 mcg/kg IV over 10 minutes, followed by 0.125-0.75 mcg/kg/min infusion

**Push Dose Pressors**

- Epinephrine 0.5-2 mL (5-20 mcg) q2-5 minutes
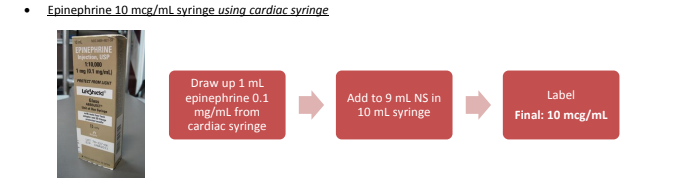

- Phenylephrine 0.5-2 mL (50-200 mcg) q2-5 minutes
  - Draw up 1 mL phenylephrine from 10 mg/mL vial
  - Add to 100 mL bag of normal saline
  - Final: 100 mLs of phenylephrine 100 mcg/mL

***(Phenylephrine “Sticks” are pre-made and are stored in the crash cart)***

**Acute Hypertension**

- Hypertensive urgency
  - Optimize home medications (restart, intensify, maximize doses)
  - If not on antihypertensives at home: (PO preferred)
    - Labetalol 200-400 mg po (or 10-20mg IV push if NPO)
    - Captopril 6.25-12.5 mg po
    - Clonidine 0.1-0.2 mg po
- Hypertensive emergency (many options: drug of choice depends on underlying cause)
- Nicardipine 5 mg/hr IV infusion titrating by 2.5 mg/hr q5-15min (max 15 mg/hr)
  - Stroke
- Labetalol 10-20 mg IV push followed by 40-80 mg q10min (max 300 mg)
  - May also start infusion at 0.5 to 2 mg/min
  - Stroke
- Nitroglycerin
  - 1-2 mg IVP bolus q3-5 minutes (max 20 mg)
  - If continuous infusion needed, range 5-200mcg/min
  - CHF; contraindicated in stroke (increase in ICP)

**Aortic Dissection**

- Esmolol 500 mcg/kg IV bolus, followed by infusion of 50-300 mcg/kg/min
  - Increase infusion by 50 mcg/kg/min every 4 minutes
  - Highly consider re-bolus each time rate is increased
- Nicardipine 5 mg/hr IV infusion titrating by 2.5 mg/hr q5-15min (max 15 mg/hr)

**Module 3 Medication Quick Reference**

**Disorders of Bleeding**

****refer to DMC anticoagulation reversal guidelines for complete guidance***

**Intracranial Hemorrhage and Anticoagulation Reversal (Warfarin)**

- Vitamin K: 10 mg IVPB q12h
- Prothrombin complex concentrate (K Centra):
  - INR 2-4: 25 U/kg (max 2500U)
  - INR 4-6: 35 U/kg (max 3500U)
  - INR >6: 50 U/kg (max 5000U)
  - If ICH on apixaban or rivaroxaban, use 50 units/kg (max 5000U)
- Mannitol: 0.5-1 g/kg
- Hypertonic NaCl: 3% infusion, 23.4% slow push: titrated to Sodium of 145-155

**Major bleeding (not ICH) on warfarin/apixaban/rivaroxaban:**

- Typically use fixed dose KCentra 1000units (+ VitK if warfarin)

**Other reversal**

- Heparin and enoxaparin: Protamine
- Dabigatran: Praxbind

***Refer to DMC anticoagulation guidelines for specific reversal/dosing for anticoagulant agents*

**Traumatic Bleeding**

- TXA
  - Loading dose: 1 g over 10 minutes
  - Infusion: 1 g over 8 hours

**Pharmacology for Endocrine and Immune Disorders**

**Acute Angle Closure Glaucoma**

- Timolol: 1 drop 0.5-1.0%
- Apraclonidine/brimonidine: 1 drop 0.2%
- Pilocarpine: 1 drop 1-2%
- Acetazolamide: 500 mg IV
- Mannitol: 1-2 g/kg IV over 45 minutes (500 mL of 20%)

**DKA**

- Potassium
  - K <3.3: Give KCl 10-20 mEq/h until >3.3
  - K 3.3 - 5.2: Give KCl 20-40 mEq in each 1L of IVF to keep >4.5
  - K >5.2: Do not use replacement protocol. Check K Q2h
    - Do not begin insulin therapy at all until K >3.5
- Insulin
  - IV infusion (0.1-0.14 units/kg/hr)
  - IV Bolus (0.1 units/kg/hr) + IV infusion (0.1 units/kg/hr) → not as often recommended
    - If the serum glucose has not decreased by 50-75 mg/dL from the initial value in the first hour, consider doubling the insulin therapy
  - Once CBG <250, reduce insulin to 0.02-0.05 units/kg/hr to maintain CBG 150-250 mg/dL until DKA resolves
- Fluids
  - 15-20 mL/kg/h over the first hour *THEN*
  - If corrected serum Na+ normal/high → 0.45% NaCl 250-500 mL/hr
  - If corrected serum Na+ low → 0.9% NaCl 250-500 mL/hr
  - Once CBG <250 change to D5W/0.45% NaCl 150-250 mL/hr

**Thyroid Storm**

- Propranolol
  - 60-80 mg PO q4h
  - 0.5-1.0 IVP over 10 min with continuous cardiac monitoring; repeat 1-3mg every few hours
- PTU
  - 500-1000 mg PO loading dose *THEN*
  - 200-250 mg PO q4h
    - May be given via NGT or rectally if pt cannot tolerate PO
- Dexamethasone 2mg IV

**Myxedema Coma**

- Levothyroxine 200-400 mcg IV then 50-100 mcg daily
- Triiodothyronine 5-20 mcg then 2.5-10 mcg q8hr
- Hydrocortisone 100 mg IV q8hr until exclusion of adrenal insufficiency

**Anaphylaxis**

- Epinephrine: 0.3-0.5 mg IM (1:1000) q5min, (peds 0.01mg/kg)
- Albuterol: 5 mg x3 (peds 2.5mg children <7)
- Diphenhydramine: 50 mg IVP (peds 1-2 mg/kg)
- Famotidine: 20 mg IV (peds 0.25 mg/kg)
- Methylprednisolone: 125 mg IVP (peds 1-2 mg/kg)

**Module 4 Medication Quick Reference**

**Infectious Disease Disorders**

****Refer to DMC guidelines for complete guidance***

*****Antibiotics may require renal adjustment based on CrCl***

**SSTI**

- Mild: Cephalexin 500mg PO (non-purulent); Bactrim DS q12H (purulent/abscess)
- Moderate/severe: Cefazolin 2gm q8h (non-purulent); Vancomycin PTD (purulent/abscess)

**Necrotizing Fasciitis**

- Piperacillin-Tazobactam: 4.5 g IV q6h (PsA/G(-), Anaerobic)
- Linezolid: 600 mg IV q12h (MRSA/G(+) with toxin reducing effects)

**Dog Bite *(need to cover mouth anaerobes)***

- Augmentin: 875 mg/125 mg PO q12h 7-10 days
- Unasyn: 3 g IV q6h 7-10 days
- If penicillin allergy:
  - Doxycycline 100mg PO bid *OR*
  - Moxifloxacin 400 mg PO qd

**Rabies Prophylaxis**

- Rabies vaccine: 1mL IM injection given days 0, 3, 7, 14
- Rabies immunoglobulin at 20 U/kg subcutaneously administered around site of bite

**HIV Prophylaxis**

- Truvada [Tenofovir (TDF) 300 mg with Emtricitabine (FTC) 200 mg] once daily + Raltegravir (RAL) 400 mg twice daily OR dolutegravir (DTG) 50 mg daily for 28 days

**Pneumonia**

- DRIP score < 4:
  - Non-ICU admission
    - Ceftriaxone 1-2g Q24 + Azithromycin 500mg PO/IV Q24 *OR* Doxycycline 200mg PO/IV x1, followed by 100mg Q12
  - ICU admission
    - Ceftriaxone 1-2g Q24 + Azithromycin 500mg PO/IV Q24 + Vancomycin pharmacy to dose
- DRIP score >/= 4:
  - Non-ICU admission
    - Cefepime 2g Q8H + Azithromycin 500 mg PO/IV Q24 + Vancomycin pharmacy to dose
  - ICU admission
    - Cefepime 2g Q8H + Azithromycin 500 mg PO/IV Q24 + Tobramycin + Vancomycin pharmacy to dose

**UTI**

- Asymptomatic Bacteriuria: No treatment unless pregnant, undergoing urologic procedure, and <1 month post-renal transplant
- Uncomplicated cystitis
  - Nitrofurantoin 100mg PO q12h X 5 days
  - Bactrim DS PO q12h X 3 days
  - Ceftriaxone 1gm IV q24h X 5 days
- Pyelonephritis/Complicated
  - Ceftriaxone 1-2g IV daily X 7 days (preferred inpatient)
  - Bactrim DS PO q12h X 7 days (preferred outpatient)
  - Ciprofloxacin 500mg q12h X 5-7 days
  - Risk factors for Abx resistance: Amikacin PTD X 7 days
- Pregnant females
  - Cephalexin 500 mg PO bid 3-7 days

**Septic Shock**

- Norepinephrine 0.1 mcg/kg/min infusion titrated to goal parameters (i.e. MAP, SBP, UOP) (max 3.3 mcg/kg/min)
- Consider adding vasopressin 0.03 units/min if escalating doses of norepinephrine

**Toxic Shock Syndrome**

- Clindamycin: 900 mg IV q8h *AND*
- Oxacillin or Nafcillin: 2 g IV q4h

**Toxicology**

**Beta Blocker Toxicity/Calcium Channel Blocker Toxicity**

- Atropine: 0.5-1.0 mg IVP q3-5 min or infusion at 0.03-0.04 mg/kg
- Calcium → increase inotropy
  - Calcium chloride (1 g of 10% solution [10 ml]) slow IVP via CVC
  - Calcium gluconate (30 ml of 10% solution)
- Dextrose → to prepare for high-dose insulin therapy
  - If CBG <200 mg/dL, provide bolus (0.25 gm/kg) in appropriate concentration (D10W, D25W, D50W) based on the fluid status
  - Infusion: 0.15 - 0.5 gm/kg/hr titrated to euglycemic status
    - Monitor the CBG every 30 minutes for first four hours, then every 1-2 hours
- Rapid Acting Insulin (Regular, Humolog) → improves inotropy
  - Bolus: 1 U/kg
  - Infusion: 0.5 - 1.0 U/kg/hr, titrated up to 8-10 U/kg/hr
    - Titrate infusion to blood pressure. Do not decrease or stop the insulin drip due to hypoglycemia, instead bolus more dextrose in this scenario
- Vasopressors → improves inotropic and chronotropic activity
  - Epinephrine: 0.06-1.2 mcg/kg/min
  - Norepinephrine: 0.01-3.3 mcg/kg/min

**Acetaminophen Toxicity**

- Activated charcoal 1 g/kg po (max dose 50 g)
- N-acetyl cysteine (NAC):
  - Loading dose: 150 mg/kg IV over 1 hour
  - Infusion: 15 mg/kg/hr over following 24 hours
  - If oral administration is tolerated: 140 mg/kg PO load, followed by 70 mg/kg Q4h doses over next 24h

**Opioid Toxicity**

- Naloxone:
  - IV: 0.4-2.0 mg starting dose
    - If chronic user, consider the minimal effective dose at 0.04 mg IV to prevent acute withdrawal
  - Intranasal: 0.4-1.0 mg/mL
  - Infusion: give ⅔ of the “wake up dose” per hour
    - given if pt responded to first dose and required repeat administration

**Salicylate Toxicity**

- Activated charcoal 1 g/kg po (max dose 50 g)
- Sodium bicarbonate
  - Bolus: 1-2 mEq/kg IV
  - Infusion: 150 mEq sodium bicarbonate in 1L D5W at 250 mL/hr (goal pH 7.45-7.55)

**Cholinergic Toxicity**

- Atropine: 2-5 mg IV doubled every 3-5 min until symptoms improve
- Pralidoxime: 30 mg/kg IV bolus *THEN* 8 mg/kg IV infusion
- Benzodiazepines:
  - Midazolam 5-10 mg IM/IV
  - Lorazepam 2-4 mg IV

**Anticholinergic Toxicity**

- Benzodiazepines
  - Midazolam 5-10 mg IM/IV
  - Lorazepam 2-4 mg IV
- Physostigmine: 0.5-2mg IV

**TCA Toxicity**

- Sodium bicarbonate
  - Bolus: 2-4 amps, if no response repeat after 5 minutes.
  - Infusion: 150 mEq sodium bicarbonate in 1L D5W at 250 mL/hr (goal pH 7.45-7.55)
- Benzodiazepines
  - Midazolam: 5-10 mg IM/IV
  - Lorazepam: 2-4 mg IV
- Vasopressors
  - Norepinephrine: 0.01-3.3 mcg/kg/min IV
  - Phenylephrine: 0.5-9 mcg/kg/min IV

**Methanol Toxicity**

- Fomepizole: 15 mg/kg IV over 30 minutes, *THEN* 10 mg/kg q12hr for 4 doses *THEN* increase back to 15mg/kg q12h (if continued) until level <20
- Folate: 50 mg IV q6h

**Ethylene Glycol Toxicity**

- Fomepizole: 15 mg/kg IV over 30 minutes, *THEN* 10 mg/kg q12hr for 4 doses *THEN* increase back to 15mg/kg q12h (if continued) until level <20
- Thiamine: 100 mg IV q6h x2 days
- Pyridoxine: 50 mg q6h x2 days
- Magnesium: 2g IV x1

**Isopropyl Alcohol Toxicity**

- Supportive Care
